# Insights into motor impairment assessment using myographic signals with artificial intelligence: a scoping review

**DOI:** 10.1007/s13534-025-00483-7

**Published:** 2025-06-05

**Authors:** Wonbum Sohn, M. Hongchul Sohn, Jongsang Son

**Affiliations:** 1 Department of Biomedical Engineering, Newark College of Engineering, New Jersey Institute of Technology, 323 Dr Martin Luther King Jr Blvd, Newark, NJ 07102 USA; 2 Department of Physical Therapy and Human Movement Sciences, Northwestern University, 645 N. Michigan Ave, Chicago, IL 60611 USA

**Keywords:** Machine learning, Deep learning, Clinical assessment, Measurement modalities

## Abstract

Myographic signals can effectively detect and assess subtle changes in muscle function; however, their measurement and analysis are often limited in clinical settings compared to inertial measurement units. Recently, the advent of artificial intelligence (AI) has made the analysis of complex myographic signals more feasible. This scoping review aims to examine the use of myographic signals in conjunction with AI for assessing motor impairments and highlight potential limitations and future directions. We conducted a systematic search using specific keywords in the Scopus and PubMed databases. After a thorough screening process, 111 relevant studies were selected for review. These studies were organized based on target applications (measurement modality, measurement location, and AI application task), sample demographics (age, sex, ethnicity, and pathology), and AI models (general approach and algorithm type). Among various myographic measurement modalities, surface electromyography was the most commonly used. In terms of AI approaches, machine learning with feature engineering was the predominant method, with classification tasks being the most common application of AI. Our review also noted a significant bias in participant demographics, with a greater representation of males compared to females and healthy individuals compared to clinical populations. Overall, our findings suggest that integrating myographic signals with AI has the potential to provide more objective and clinically relevant assessments of motor impairments.

## Introduction

Motor impairments are prevalent in various clinical conditions such as stroke [1], spinal cord injury (SCI) [2], cerebral palsy (CP) [3], amyotrophic lateral sclerosis (ALS) [4], myopathy [5, 6], neuropathy [5, 6], multiple sclerosis (MS) [7], and Parkinson’s disease (PD) [8], significantly affecting patients’ ability to perform daily tasks and independence, and thus their quality of life. Objective assessment of motor impairments is crucial for enabling tailored care (diagnosis, treatment, and intervention) for such wide range of clinical population in need [9]. However, current clinical assessments often rely on subjective evaluations, leading to several limitations such as inter-rater variability [10] and ceiling effect [11]. On the other hand, laboratory-based quantification of motor impairment is not readily translatable to clinical settings, mainly due to practical barriers in transferring the technical resources (e.g., equipment, knowledge, and skills) required for acquisition, processing, and analysis/interpretation of the data collected [12]. There is an urgent need for objective clinical tools that can provide timely and precise assessments of motor impairments for effective intervention and precise medicine.

Recent advances in sensor and artificial intelligence (AI) technologies offer promising avenues for quantitative assessment of motor impairments in the real-world (e.g., clinical and/or daily setting) [13, 14]. As such, a large volume of studies in the past decade has focused on integrating AI with sensor-based measurements of human movement to recognize pattern/activity [15–23] or user intent [24–26], detect disease symptoms or adverse events [27–32], or provide bio-feedback during movement training/therapy [33]. Among the many sensor modalities used, inertial measurement units (IMUs) have dominantly been adopted, mainly owing to their compact size, low cost, ease of use (e.g. placement), and reliable performance [34, 35]. However, IMUs only measure motion, solely derived from kinematic parameters (i.e., translational acceleration, rotational speed, and orientation in space), and provide no information about the muscle activity or contraction that caused the movement, which often can result in little to no observable “motion” (i.e., isometric). For most, if not all, motor impairments, however, muscle activity is one of the most critical pieces of information for a comprehensive understanding of the underlying physiological mechanisms, as it is the ultimate manifestation of how the nervous system controls the physical part of the human body (e.g., limb segment).

**Fig. 1:**
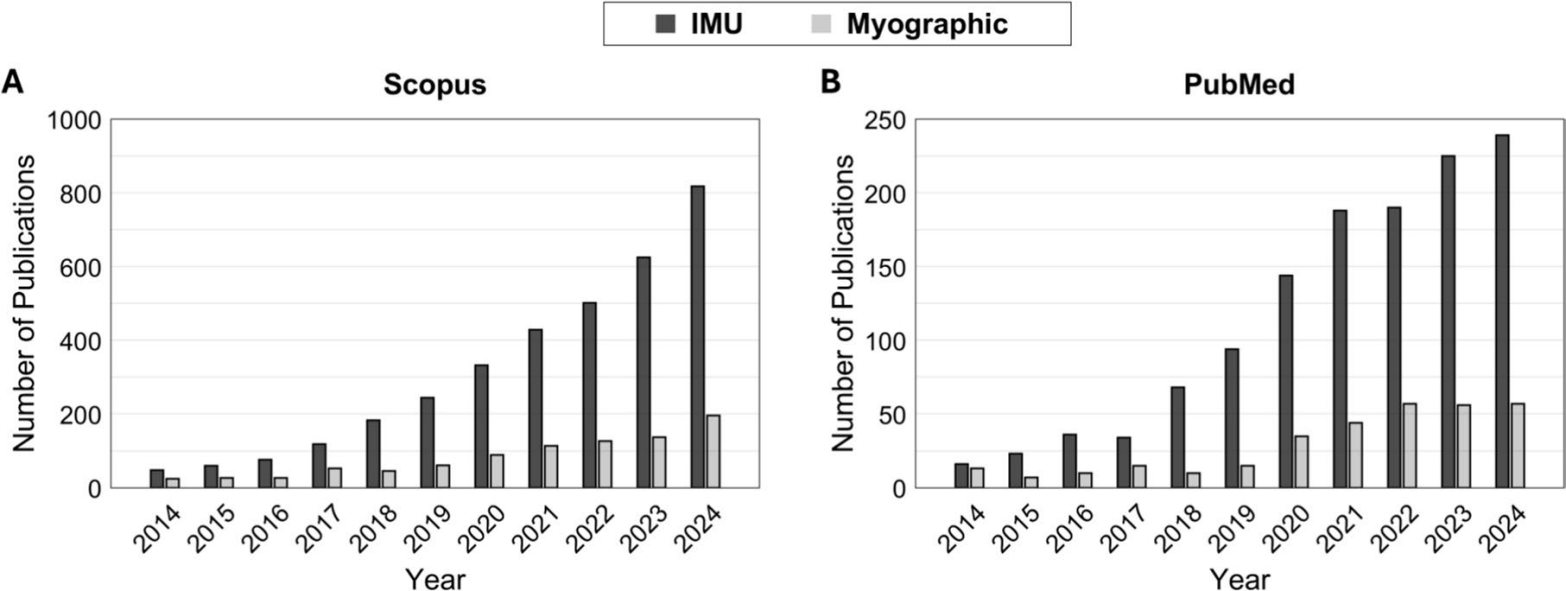
The number of publications from 2014 to 2024 in the Scopus (**A**) and PubMed (**B**) databases. The publications are categorized by measurement modality used: inertial measurement units (IMUs) versus myographic signals. While there has been an evident rise in the number of research using each IMU and myographic signals, myographic signals have consistently received less attention than IMUs

We postulate that myographic signals—physiological activities measured from muscles—confer more than what IMU can offer, especially for motor impairment assessment. For example, myographic signals reveal complex physiological patterns such as coactivation [36, 37], fatigue [38–40], response to various types of sensorimotor stimulus [41, 42], motor unit recruitment [43], and the potential source (e.g., brain areas) governing the neural drive to the muscles [44, 45]. These insights cannot be captured through kinematic measurements alone and are crucial for understanding the pathophysiological mechanisms underlying abnormal movement patterns in clinical populations. By leveraging the information acquired with myographic signals, clinicians can detect subtle changes in muscle function and identify biomarkers that reflect the status of neuromuscular diseases, allowing for an evaluation of muscle function in real-world settings. Despite their significant potential, the technical challenges involved in acquiring, processing, and analyzing myographic signal (signal-to-noise ratio, location dependence, motion artifact, etc.) hinder the widespread adoption in clinical settings [46, 47]. Given the premise that AI is specialized in automated data processing/analysis and making prediction/inference based on potential patterns underlying large/complex set of signals, the AI-powered motor impairment assessment using myographic signals can address these limitations and offer a promising tool that provides more objective, precise, and clinically relevant information and insights for motor impairment assessment in clinical settings. Despite its promise, the use of myographic signals with AI models has received relatively little attention compared to IMU-based approaches, as evidenced by our preliminary literature search in Scopus and PubMed databases (Fig. 1).

In the light of this knowledge gap, the purpose of this study is to provide a comprehensive overview of current use of myographic signals with AI for motor impairment assessment in the aspects of target application (e.g., classification, regression), sample demographics (e.g., age, sex, ethnicity, pathology), and AI model (e.g., machine learning, deep learning) and to discuss potential limitations and future directions for each of the above aspects.

## Methods

**Fig. 2:**
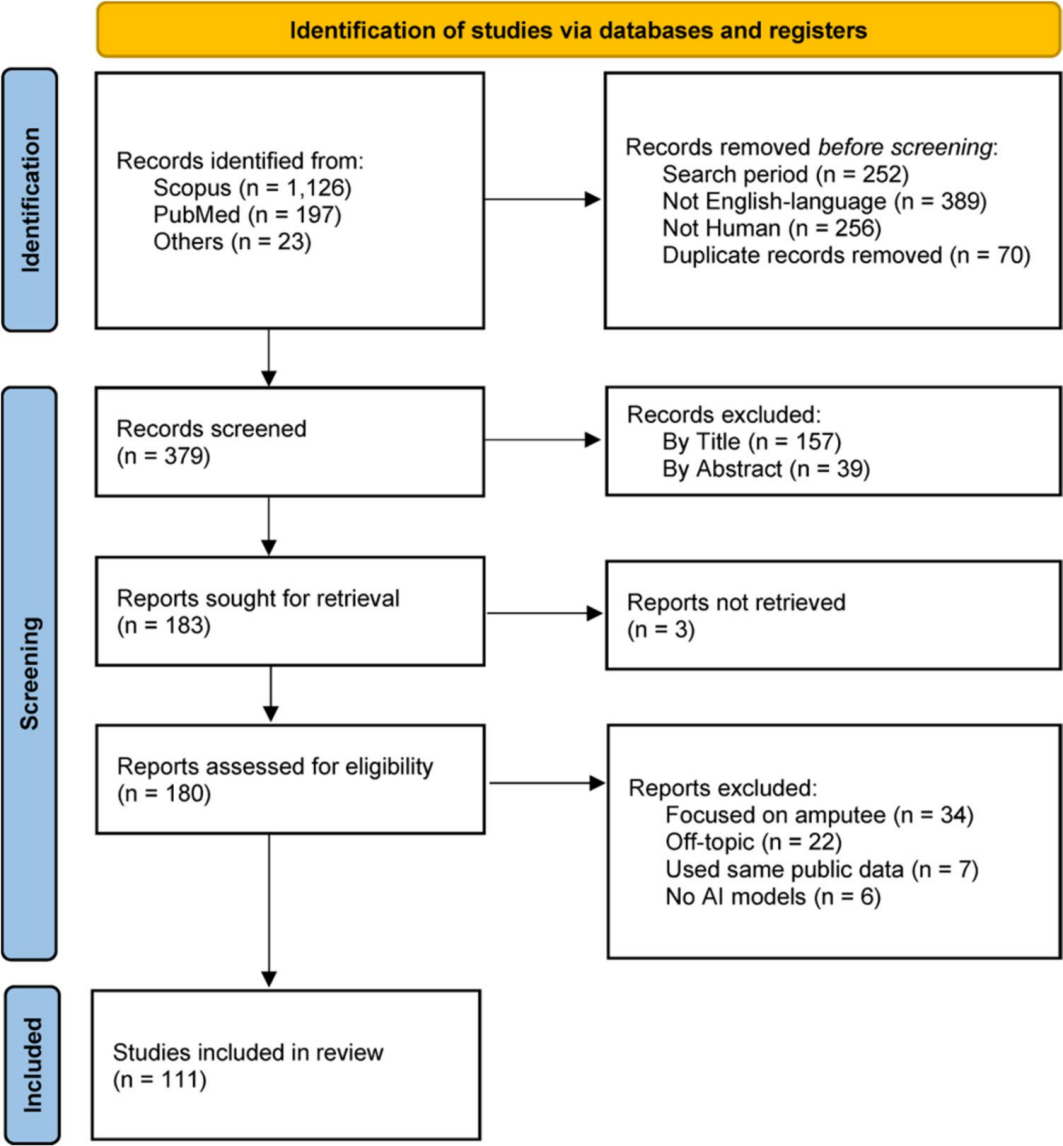
Overview of the PRISMA process for conducting a literature search, screening articles, and including relevant studies for this scoping review

A comprehensive literature search was conducted using Scopus and PubMed databases, followed by a study selection process in general accordance with the Preferred Reporting Items for Systematic Reviews and Meta-Analyses (PRISMA) guideline. The initial search was performed using the following keyword combinations: ("AI" OR "artificial intelligence" OR "machine learning" OR "deep learning" OR "neural network") AND ("medical" OR "clinical" OR "patient" OR "assessment" OR "monitoring" OR "diagnosis" OR "tracking" OR "impairment") AND ("motor" OR "movement" OR "force" OR "torque" OR "strength" OR "kinematics") AND ("myogram" OR "myography" OR "EMG" OR "MMG" OR "FMG" OR "OMG" OR "SMG" OR ("ultrasound" AND "muscle")). The initial search results were further filtered with the following criteria: (1) restricting to works published within the last decade (2014–2024); (2) restricting to English-written, peer-reviewed journal article; and (3) excluding animal works. To satisfy the criteria, the database-specific search strategies were implemented as follows. In PubMed, the Advanced Search function was used with the ‘Title/Abstract’ field selected, and the aforementioned keyword combinations were applied. To refine the results in accordance with the predefined criteria, filters were set as follows: publication years from 2014 to 2024, article language as English, and species limited to Humans. In Scopus, the search was conducted by selecting ‘Article title, Abstract, Keywords’ under the Search within field, using the same keyword combinations. Subsequently, the following filters were applied: publication years from 2014 to 2024, document type as Article, language as English, and keywords limited to Human and Humans. Finally, each study was manually screened, sequentially in the order by title, abstract, then full-text, based on the following criteria: (1) targeting clinical application; (2) measuring myographic signals; (3) utilizing machine learning and deep learning models; and (4) reporting results using myographic signals only. Additionally, relevant articles that satisfied the inclusion criteria were further identified through a manual search. The PRISMA flow diagram is shown in Fig. 2.

The selected studies were reviewed in detail, specifically focusing on the following scopes:

- *Target application.* The selected studies were categorized into measurement modality, location (e.g., target muscle or joint), and purpose of the AI model/application. From detailed review of the selected studies with respect to this scope, we sought to determine whether the use of myographic signals indeed can provide more tailored insights into assessing motor impairment and/or allow for better performance of AI models compared to other measurement modalities such as IMU.
- *Sample demographics*. Participant population in the selected studies were analyzed in terms of the portions between healthy individuals versus patients, males versus females, and ethnicities or origins of the populations based on study authors’ affiliations. In addition, we determined the age distribution among the healthy and patient groups in the studies where the mean and standard deviation values were reported, as well as the relationship between the number (i.e., absolute count) of male versus female participants. From this scope, we sought to determine whether there exist any potential biases that may hinder the generalization of the developed/applied AI models, accounting for the heterogeneity in a particular clinical target population, or even broader populations in general.
- *AI model*. The AI models used in the selected studies were categorized into general types of approach (i.e., machine learning with feature engineering, deep learning with feature engineering, deep learning without feature engineering) and specific algorithm. From this scope, we sought to determine whether the current approaches have potential implications for generalizability to diverse contexts of application.

In particular, we prioritized the studies that included patient populations for the above analyses in Target Application and AI Model.

## Results

The initial literature search yielded a total of 1,346 studies. After restricting the search period from 2014 to 2024, 1,094 studies remained. Limiting the search to English-language articles further reduced the number to 705. Next, filtering for studies that focused on human subjects resulted in a selection of 449 studies. After removing duplicates, 379 studies were left. Based on the inclusion criteria, title screening reduced this number to 222, and abstract screening further narrowed it down to 183. Finally, after a full-text review, 111 studies were selected for the final analysis (Fig. 1). Out of the selected 111 studies, 64 studies were conducted with only healthy participants [38–40, 46, 48–107], and 47 studies included data collected from patients [15–33, 108–135].

We summarized the number and age of participants in the control (healthy) group from studies that included patient groups, as well as the number and age of participants in each patient group. If available, the total number of female and male participants was also summarized. Additionally, we extracted details on the model inputs and outputs, the AI algorithms used, and the performance metrics reported in each study (Tables 1 and 2). Table 1 presents studies in which the AI models performed classification tasks, while Table 2 summarizes those involving regression tasks. In the following sections, we provide a detailed review of the selected studies with respect to each of the three scopes and describe the results of the analysis proposed.

**Table 1:** Summary of studies employing AI models for classification tasks

Study	Participants	Models	Performance
Gokgoz et al. (2014)	Control: 10 (21–37) Myopathy: 8(19–63) ALS: 8 (35–67) Female:Male = 10:15	Input: MUSIC of iEMG Output: Disorder (Healthy, Myopathic, ALS) Algorithms: KNN, NN, SVM	Accuracy: 82.11% (KNN), 90.02% (NN), 92.55% (SVM) Myopathic sensitivity: 85.00% (KNN), 82.00% (NN), 91.00% (SVM) ALS sensitivity: 95.30% (KNN), 95.67% (NN), 96.33% (SVM) Specificity: 66.00% (KNN), 93.00% (NN), 90.30% (SVM)
Liu et al. (2014)	Control: 0 SCI: 9 (31–62) Female:Male = 3:6	Input: AR, MAV, RMS, SSC, ZC of HD-sEMG Output: Grasp patterns and hand state Algorithms: KNN	Accuracy: 95%
Priyadharsini et al. (2015)	Control: 15 (22–42) Myopathy: 1 (75) Neuropathy: 11 (55–65) Female:Male = 18:9	Input: DWT, WPT-based statistics of sEMG Output: Disorder (Healthy, Myopathic, Neuropathic) Algorithms: NN	Accuracy: 100%
Ramos-Murguialday et al. (2015)	Control: 0 Stroke: 48 (55.01 ± 11.3)	Input: WL of sEMG Output: Movement intention Algorithms: NN	Accuracy: 47.09 ± 15.10% (Affected), 65.82 ± 14.81% (Unaffected)
Subasi (2015)	Control: 7 Myopathic: 7 Neurogenic: 13 Female:Male = 12:15	Input: DWT-based statistics of iEMG Output: Disorder (Healthy, Myopathic, Neurogenic) Algorithms: SVM	Accuracy: 97.00%, Myopathy sensitivity: 98.25%, Neurogenic sensitivity: 99.00%, Specificity: 93.75%
Gandolla et al. (2016)	Control: 8 (25–26) Stroke: 2 (48, 50) Female:Male = 5:5	Input: sEMG Output: Hand grasp Algorithms: NN	Accuracy: 76 ± 14%
Jordanic et al. (2016)	Control: 0 SCI: 9 (47 ± 18) Female:Male = 5:4	Input: Intensity and center of gravity of HD-EMG maps, Intensity of a single differential EMG channel HD-sEMG Output: Identification of task and effort level Algorithms: LDA	Accuracy: 98.7% (Task intention), 98.8% (Force intention)
Barmpakos et al. (2017)	Control: 9 (53.7 ± 13.7) Myopathy: 9 (52.8 ± 6.3) Neuronal: 9 (50.6 ± 14.9) Female:Male = 10:17	Input: MAV, RMS, SSC, WE, WL, ZC, WA of sEMG Output: Disorder (Healthy, Myopathic, Neuropathic) Algorithms: KNN	Accuracy: 98.36 ± 0.79%
Artuğ et al. (2018)	Control: 4 (32–47) Myopathy: 2 (27–47) Neurogenic: 3 (39–78) Female:Male = 2:7	Input: Maximum amplitude, NP, NPO, RPOPI, Spike duration of iEMG Output: Disorder (Healthy, Myopathic, Neurogenic) Algorithms: KNN, NN, SVM	Accuracy of simulated data: 98.00% (KNN), Accuracy of EMG data: 85.00% (MLP)
Ma et al. (2019)	Control: 5 (25.7 ± 1.8) Stroke: 9 (25–75)	Input: AR, MAV, RMS, VAR, WL of sEMG Output: Trunk compensation classification Algorithms: SVM	Accuracy of lean-forward: 94.0% (Healthy), 74.8% (Stroke) Accuracy of trunk rotation: 95.8% (Healthy), 67.1% (Stroke) Accuracy of shoulder elevation: 100.0% (Healthy), 91.3% (Stroke)
Mesbah et al. (2019)	Control: 0 SCI: 11 (22, 23, 24, 24, 24, 26, 27, 31, 32, 33, 35) Female:Male = 1:10	Input: IMF, Mean and Median frequency, MPV, PV, TP of sEMG Output: Standing (Assisted, Independent) Algorithms: Bayes, DT, KNN, SVM	Accuracy: 95.3%
Nodera et al. (2019)	Control: 0 Patients: 103 (Radiculopathy, amyotro-phic lateral sclerosis, poly-myositis, muscular dystro-phy, and peripheral nerve injuries)	Input: Energy, FFT, MFCC of iEMG Output: Classified EMG resting potentials Algorithms: Bayes, Boost, Linear, SVM	Accuracy: 90.4% (IS-09 Set), 89.9% (IS-11 Set)
Nodera et al. (2019)	Control: 7 (24–58) ALS: 8 (42–71) Female:Male = 6:9	Input: SMG Output: Movement Algorithms: NN	Accuracy for training without pre-trained weights: 100% (VGG16), 100% (VGG19), 90.5% (ResNet-50), 86.6% (ResNet-152), 99.2% (Inception-v3) Accuracy for training with pre-trained weights: 100% (VGG16), 100% (VGG19), 99.8% (ResNet-50), 99.7% (ResNet-152), 100% (Inception-v3)
Alfaro-Ponce et al. (2020)	Control: 81 PD: 93	Input: sEMG Output: Movements, Gait phases Algorithms: NN	Accuracy of fivefold cross validation process: 98.08% (DifNN), 97.91% (TDNN), 96.66% (CVNN) Accuracy of generalization-regularization methods: 98.14% (DifNN), 98.60% (TDNN), 95.36% (CVNN)
Huo et al. (2020)	Control: 10 (27.6 ± 3) PD: 23 (73, 59, 61, 57, 37, 42, 43, 61, 52, 50, 41, 68, 62, 66, 54, 67, 51, 69, 71, 63, 70,53, 56) Female:Male = 13:20	Input: FFT, Mean, SD of MMG, IMU Output: Disorder (Healthy, UPDRS scores(1–3)) Algorithms: Boost, KNN, NN, RF	Sensor Comparison for PD Symptom Assessment:\ 82.7% (IMU), 81.7% (Elbow of force sensor), 82.5% (Wrist of force sensor), 81.5% (Tremors of MMG), 76.3% (Bradykinesia of MMG), 85.4% (Fusion) Performance in Identifying PD Patients versus Healthy: 96.6% (PD vs Healthy), 89% (All symptoms)
Jochumsen et al. (2020)	Control: 0 Stroke: 16 (53 ± 8) Female:Male = 1:15	Input: MAV, SSC, WL, ZC of sEMG Output: Motion Algorithms: NN	Within-session accuracy: 79 ± 12% (Day 1), 80 ± 12% (Day 2) Between-session accuracy (Day 1–2): Approximately 30%
Liew et al. (2020)	Control: 16 LBP: 33	Input: sEMG Output: Disorder (Healthy, Remission LBP, Current LBP) Algorithms: Boost	AUC: 90.4–96.7%
McDonald et al. (2020)	Control: 10 (20–28) SCI: 4 (22, 23, 30, 49) Female:Male = 2:12	Input: sEMG Output: Movement directions Algorithms: LDA	Averages of single-DOF classification accuracy: Over 99% (Able-bodied), 85.5–95.0% (SCI) Averages of multi-DOF classification accuracy: Near 90% (Able-bodied), Just above 60% (SCI)
Wang et al. (2020)	Control: 15 (48.5 ± 13.1) Stroke: 15 (52.1 ± 15.1) Female:Male = 11:19	Input: DAMV, kWAS, MAV, PCA, SD of sEMG, kinematic Output: Movement Algorithms: NN, RF, SVM	EMG: 90.74% (NN), 89.12% (RF), 89.35% (SVM) Kinematic: 88.19% (NN), 88.66% (RF), 92.82% (SVM) Fusion: 94.21% (NN), 93.89% (RF), 96.06% (SVM)
Yun et al. (2020)	Control: 2 (34, 35) SCI: 4 (34, 51, 57, 59)	Input: sEMG Output: Hand poses Algorithms: NN	Accuracy: 97.74% (H01), 97.1% (H02), 88.8% (S01), 96.99% (S02), 88.2% (S03), 62.80% (S04)
Formstone et al. (2021)	Control: 0 Stroke: 64 (66.3 ± 13.8) Female:Male = 31:33	Input: Features-based PCs of MMG, IMU (LD, Mean frequency, Mean power, MPR, NMC, PR, PSR, Skewness, SSC, TRI, VCF) Output: FMA-UE score Algorithms: Boost	Accuracy: 75% (Gross motor tasks (IMU)), 62% (Hand/Wrist motor tasks (MMG))
Ghislieri et al. (2021)	Control: 8 (38.0 ± 13.1) THA: 6 (73.8 ± 8.4) NPH: 6 (75.7 ± 6.3)	Input: sEMG Output: Muscle activation Algorithms: NN	Accuracy: 96.8 ± 4.3%
Hussain et al. (2021)	Control: 75 (mean: 77) Stroke: 48 (72.2 ± 5.6)	Input: Mean and median power frequency, Mean power, PPF, TP of sEMG Output: Disorder (Healthy, Post-stroke) Algorithms: DT, Logistic, NN, SVM	Accuracy: 65%
Meng et al. (2021)	Control: 14 Brunnstrom stage V: 7 Brunnstrom stage IV: 2 Female:Male = 10:13	Input: Mean, median, VAR of sEMG, Accelerometer, Gyroscope Output: Daily activities Algorithms: SVM	Accuracy of healthy: 90.87 ± 3.41% (sEMG), 94.54 ± 2.06% (Gyroscope), 96.43 ± 2.35% (Accelerometer), 96.53 ± 2.36% (Fusion) Accuracy of stroke: 73.77 ± 3.73% (sEMG), 75.88 ± 5.53% (Gyroscope), 94.05 ± 3.10% (Accelerometer), 94.22 ± 2.63% (Fusion) Accuracy of all: 84.09 ± 3.33% (sEMG), 87.95 ± 2.79% (Gyroscope), 95.84 ± 1.75% (Accelerometer), 96.56 ± 1.55% (Fusion) Accuracy of healthy (train) and stroke (test): 76.34 ± 3.75% (sEMG), 76.82 ± 5.55% (Gyroscope), 77.89 ± 4.81% (Accelerometer), 82.47 ± 4.18% (Fusion)
Morbidoni et al. (2021)	Control: 0 MHCP: 20 (9.3 ± 3.2) Female:Male = 9:11	Input: sEMG Output: Phase Algorithms: NN	Accuracy: 97 ± 1% (Intra-subject), 91 ± 3% (Inter-subject)
Noor et al. (2021)	Control: 0 Stroke: 11 (40, 48, 49, 50, 56, 57, 58, 59, 60, 61, 62) Female:Male = 0:11	Input: MAV, SSC, WA, WL, ZC of sEMG Output: Ankle joint movemen Algorithms: LDA, NN	Accuracy: 63.86 ± 4.3% (LDA), 67.1 ± 7.9% (NN)
Quintão et al. (2021)	Control: 20 (47.0 ± 7.0) ALS: 13 (59.0 ± 9.0) Female:Male = 23:10	Input: Coherence, Correlation, DFA, Kurtosis, LZ, Maximum Frequency, ME, MFL, Non-equispaced FFT, Power Bandwidth, PLF, Skewness, Spectral Kurtosis of sEMG Output: Disorder (Healthy, ALS) Algorithms: Bayes, Boost, DT, KNN, LDA, RF	Accuracy of left limb: 67.4 ± 14.0% (Bayes), 83.0 ± 7.1% (Boost), 79.4 ± 7.3% (DT), 67.7 ± 9.6% (KNN), 72.3 ± 11.1% (LDA), 69.0 ± 9.6% (RF) Accuracy of right limb: 68.1 ± 14.2% (Bayes), 80.6 ± 6.9% (Boost), 78.8 ± 9.1% (DT), 68.7 ± 10.6% (KNN), 70.6 ± 12.8% (LDA), 70.1 ± 8.0% (RF)
Ting et al. (2021)	Control: 0 SCI: 1 (32) Female:Male = 0:1	Input: NMF, PCA, RMS of sEMG, HD-sEMG Output: Finger Algorithms: LDA	Accuracy: 77.5% (RMS of EMG signals), 78.5% (Smoothed motor unit firing rates)
Zhou et al. (2021)	Control: 10 (20–24) Stroke: 5 (50–81) Female:Male = 6:9	Input: DAMV, MAV, SSC, VAR, ZC of sEMG, kinematic Output: Hand movement Algorithms: LDA	Accuracy of EMG: 88.71 ± 2.79% (Healthy), 83 ± 8.21% (Stroke) Accuracy of kinematic: 84.49 ± 6.77% (Healthy), 84.71 ± 4.54% (Stroke) Accuracy of feature fusion: 96.90 ± 1.81% (Healthy), 96.43 ± 3.83% (Stroke) Accuracy of decision fusion: 93.91 ± 2.57% (Healthy), 91.18 ± 5.50% (Stroke)
Dubey et al. (2022)	Control: 11 (21–44) Myopathy: 8 (19–63) ALS: 9 (35–67) Female:Male = 12:16	Input: CPP, EMD, HT of iEMG Output: Disorder (Healthy, Myopathy, ALS) Algorithms: DT, NN, SVM	Accuracy: 99.53%, Sensitivity: 99.25%, Specificity: 99.60%
Haque et al. (2022)	Control: 6 (52.2 ± 6.9) DN: 6 (56.0 ± 8.5) DFU: 9 (55.6 ± 6.0) Female:Male = 15:6	Input: AC, AR, Complexity, LMAV, Mobility, Moment, NSV, Skewness, SSC, WA, WL, ZC of sEMG, GRF Output: Disorder (Healthy, DN, DFU) Algorithms: BDC, DAC, ECM, KCM, KNN, LCM, NBC, SVM	Accuracy: 96.18% (EMG), 98.68% (GRF)
Leone et al. (2022)	Control: 0 Sarcopenia: 32 (Male: 63.95 ± 5.54, Female: 65.62 ± 7.30) Female:Male = 13:19	Input: AAC, AIF, Integrated EMG, MAV, Modified MAV, RMS, SSC, SSI, VAR, WA, ZC of sEMG Output: Sarcopenia confidence levels (1–3) Algorithms: Boost, DT, KNN, LR, NB, NN, RF, SVM	Accuracy: 93.6% (Boost), 86.7% (DT), 93.3% (KNN), 90% (LR), 82.8% (NB), 90% (NN), 86.7% (RF), 96.7% (SVM)
Li et al. (2022)	Control: 0 Stroke: 10 (38, 41, 62, 63, 68, 70, 72, 82, 82, 87) Female:Male = 3:7	Input: MAV, Mean and Median frequency, RMS, VAR, WL of sEMG Output: Motion intention Algorithms: NN	Accuracy: 93.11 ± 1.39%
Song et al. (2022)	Control: 0 Stroke: 10 (58.3 ± 18.09) Female:Male = 2:8	Input: AR, MAV, SSC, WL, ZC of sEMG, FMG, IMU Output: Hand movements Algorithms: DT, KNN, LDA, RF, SVM	Accuracy: 69.6% (EMG), 63.2% (FMG), 47.8% (IMU), 81.0% (Fusion)
Hou et al. (2023)	Control: 0 PD: 12	Input: sEMG, Accelerometer Output: Freezing of gait Algorithms: NN	AUC: 0.78 (EMG), 0.71 (Accelerometer), 0.75 (EEG), 0.85 (Fusion)
Kumar et al. (2024)	Control: 49 (29–49.5) Sarcopenia: 44 (27.5–49.8) Female:Male = 49:44	Input: Kurtosis, MAV, Mean and Median frequency, Peak frequency, RMS, VAR, SD, Skewness, SSC, WL, ZC of sEMG Output: Disorder (Healthy, Risk of sarcopenia) Algorithms: Bayes, Boost, KNN, NN, RF	Without EMD: 83% (Normal walking), 85% (Fast walking), 81% (Standard squat), 79% (Wide squat) With EMD: 88% (Normal walking), 89% (Fast walking), 81% (Standard squat), 80% (Wide squat)
Li et al. (2024)	Control: 45 (68.6 ± 5.8) Sarcopenia: 48 (75.3 ± 7.7) Female:Male = 60:33	Input: CWTP, Integrated EMG, KCWT, MAV, RMS, SSC, WE, WL, ZC of sEMG Output: Disorder (Healthy, Sarcopenia) Algorithms: Boost, RF, SVM	Accuracy: 70 ± 6% (Boost), 70 ± 6% (RF), 67 ± 4% (SVM), 73 ± 7% (Voting)
Simpetru et al. (2024)	Control: 13 (25.9 ± 2.8) SCI: 8 (34, 34, 38, 39, 39, 41, 44, 57)	Input: MAV, RMS, SSC, WL, ZC of HD-sEMG Output: Hand movements Algorithms: LDA, NN, Ridge	Accuracy: 99.8% (Healthy), 98.3% (SCI)
Spieker et al. (2024)	Control: 11 (34.2 ± 6.9) MS: 11 (55.9 ± 9.2) Female:Male = 10:12	Input: Single-pulse, Differential signal, Single- and double-pulse of sEMG, MMG, IMU Output: Muscle responses Algorithms: LDA, RF, SVM	Accuracy: 87 ± 8% (No, Muscle or reflex), 74 ± 11% (No, Reflex, Direct muscular)

*AAC, average amplitude change; AC, amplitude change; AIF, averaged instantaneous frequency; ALS, amyotrophic lateral sclerosis; AR, autoregressive model coefficient; AUC, area under the curve; BDC, binary decision classification tree; CPP, complex plane plot; CVNN, complex-valued neural network; CWTP, Continuous wavelet transform power; DAC, discriminant analysis classifier; DAMV, difference absolute mean value; DFA, detrended fluctuation analysis; DFU, diabetic neuropathy with ulceration; DifNN, differential neural network; DN, diabetic neuropathic; DOF, degree-of-freedom; DT, decision tree; DWT, discrete wavelet transform; ECM, ensemble classification model; EMD, empirical mode decomposition; FFT, fast fourier transform; FMA-UE, Fugl-Meyer assessment of the upper extremity; GRF, ground reaction forces; HT, hilbert transform; IMF, instantaneous median frequency; KCM, kernel classification model; KCWT, kurtosis of continuous wavelet transform; KNN, k-nearest neighbor; kWAS, k-weighted angular similarity; LBP, low back pain; LCM, Linear classification model; LD, Log detector; LDA, linear discriminant analysis; LMAV, log of the mean absolute value; LR, logistic regression; LZ, Lempel–Ziv coefficient; MAV, mean absolute value; ME, multiscale entropy; MFCC, Mel-frequency cepstral coefficients; MFL, maximum fractal length; MHCP, mild hemiplegic cerebral palsy; MPR, myopulse percentage rate; MPV, maximum power variability; MS, multiple sclerosis; MUSIC, multiple signal classification; NB, naive bayes; NBC, naive bayes classifier; NMC, normalized median crossing; NMF, nonnegative matrix factorization; NN, neural network; NP, number of peaks; NPH, normal pressure hydrocephalus; NPO, number of peaks outside the activity corridor; NSV, nonlinear scaled value; PCA, principal component analysis; PD, Parkinson’s disease; PLF, phase locking factor; PPF, peak power frequency; PR, power ratio; PSR, power spectrum ratio; PV, pattern variability; RF, random forest; RMS, root mean square; RPOPI, ratio of power outside the activity corridor to power inside the activity corridor; SCI, spinal cord injury; SD, standard deviation; SSC, slope sign changes; SSI, simple square integral; SVM, support vector machine; TDNN, time-delay neural network; THA, total hip arthroplasty; TP, total power; TRI, trapezoidal rule integration; UPDRS, unified Parkinson’s disease rating scale; VAR, variance; VCF, variance of central frequency; WA, willison amplitude; WE, wavelet energy; WL, waveform length; WPT, wavelet packet transform; ZC, zero crossings

**Table 2:** Summary of studies employing AI models for regression tasks

Study	Participants	Models	Performance
Li et al. (2016)	Control: 3 SCI: 3 (24, 36, 48)	Input: MAV of sEMG Output: Joint torque Algorithms: Kalman, NN	RMSEs: 2.10 ± 2.22 (Kalman), 1.13 ± 0.87 (NN) NRMSEs: 15.48 ± 6.67 (Kalman), 10.15 ± 6.40 (NN) VAF: 75.97 ± 12.65 (Kalman), 85.73 ± 7.31 (NN)
Li et al. (2017)	Control: 16 (36.25 ± 15.19) Stroke: 18 (55.28 ± 12.25) Female:Male = 13:21	Input: MDP, TD, EMG power distribution of sEMG, IMU Output: Correlation Algorithms: LASSO, MDS, NMF, PCA	DTW: 0.3563 (EMG), 0.7977 (IMU), 0.7781 (Fusion) PCC: 0.6672 (EMG), 0.8736 (IMU), 0.8780 (Fusion)
Dzulkifli et al. (2018)	Control: 0 SCI: 8	Input: RMS, ZC of MMG Output: Knee torque Algorithms: NN	RMS features: 79 ± 14%, RMS + ZC features: 86 ± 11%
Wang et al. (2020)	Control: 15 (48.5 ± 13.1) Stroke: 15 (52.1 ± 15.1) Female:Male = 11:19	Input: DAMV, kWAS, MAV, PCA, SD of sEMG, kinematic Output: Correlation Algorithms: NN, RF, SVM	EMG:.69, Kinematics: − 0.72, Fusion: − 0.87
Castiblanco et al. (2021)	Control: 23 Stroke: 4	Input: MAV, RMS, SSC, WL, ARC, Mean frequency, Mean power, Median frequency, SM of sEMG Output: Velocity Algorithms: Fuzzy, NN	Error percentage of PIP joint: 18% (finger II), 17% (finger III), 19% (finger IV), 20% (finger V) Error percentage of DIP joint: 5.6% (finger II), 3.9% (finger III), 4.4% (finger IV), 3.9% (finger V)
Morbidoni et al. (2021)	Control: 0 MHCP: 20 (9.3 ± 3.2) Female:Male = 9:11	Input: sEMG Output: Time Algorithms: NN	Heel-strike: 14.8 ± 3.2 (Intra-subject), 18.3 ± 4.6 (Inter-subject) Toe-off: 17.6 ± 4.2 (Intra-subject), 22.5 ± 5.6 (Inter-subject)
Ye et al. (2021)	Control: 0 Stroke: 29 (58.7 ± 8.3) Female:Male = 6:23	Input: MAV, ZC, SSC, RMS, WL of sEMG Output: Correlation Algorithms: NN	Shoulder/elbow: 0.9, Wrist/hand: 0.93
Lu et al. (2023)	Control: 15 Stroke: 2 (58, 61)	Input: AEMG of sEMG Output: Next AEMG value Algorithms: NN	MAPE: 0.68 ± 0.10 (RF), 0.85 ± 0.07 (BF), 0.43 ± 0.07 (TA), 0.84 ± 0.11 (PB) RMSE ($${10}^{-5})$$: 7.00 ± 1.75 (RF), 5.47 ± 1.38 (BF), 8.23 ± 2.08 (TA), 10.9 ± 2.7 (PB)
George et al. (2024)	Control: 10 (7.2 ± 2.4) SCI: 11 (7.1 ± 2.7) Female:Male = 8:13	Input: AUC, Maximum value, Minimum value, Average value, PPA, LMV, RMS, VLSCP, Center of max 30% of data, Power spectrum, Spectral centroid of sEMG Output: Activation levels Algorithms: DT, NN	MAPE: 229–13,809% (MLR), 127–1459% (DT), 42–113% (NN) MSE: 115.8–2908.2 (MLR), 38.4–672.2 (DT), 67.1–910.6 (NN)
Simpetru et al. (2024)	Control: 13 (25.9 ± 2.8) SCI: 8 (34, 34, 38, 39, 39, 41, 44, 57)	Input: MAV, RMS, SSC, WL, ZC of HD-sEMG Output: Mean Euclidean distance Algorithms: LDA, NN, Ridge	Healthy: 13.6 ± 15.4 mm, SCI: 23.9 ± 23.2 mm
Wang et al. (2024)	Control: 11 KA: 11 Female:Male = 0:22	Input: sEMG Output: Knee joint angle Algorithms: NN	RMSE: 4.225 ± 1.131 (Healthy), 3.913 ± 0.839 (Abnormality)

*AEMG, average rectified EMG; ARC, auto-regressive coefficients; DAMV, difference absolute mean value; DIP, distal interphalangeal; DT, decision tree; DTW, dynamic time warping correlation; KA, knee abnormalities; kWAS, k-weighted angular similarity; LASSO, least absolute shrinkage and selection operator; LDA, linear discriminant analysis; LMV, location of the maximum and minimum value; MAPE, mean absolute percentage error; MAV, mean absolute value; MDP, motion data profile; MDS, multidimensional scaling; MHCP, mild hemiplegic cerebral palsy; MLR, multiple linear regression; MSE, mean squared error; NMF, non-negative matrix factorization; NN, neural network; NRMSEs, normalized root mean square errors; PCA, principal component analysis; PCC, Pearson’s correlation coefficient; PIP, proximal interphalangeal; PPA, peak-to-peak amplitude; RF, random forest; RMS, root mean square; RMSE, root mean square error; SCI, spinal cord injury; SD, standard deviation; SM, spectral moments; SSC, slope signal changes; SVM, support vector machine; TD, time duration; VAF, variance accounted; VLSCP, value of largest sum of contiguous points; WL, waveform length; ZC, zero crossing

### Target application

**Fig. 3:**
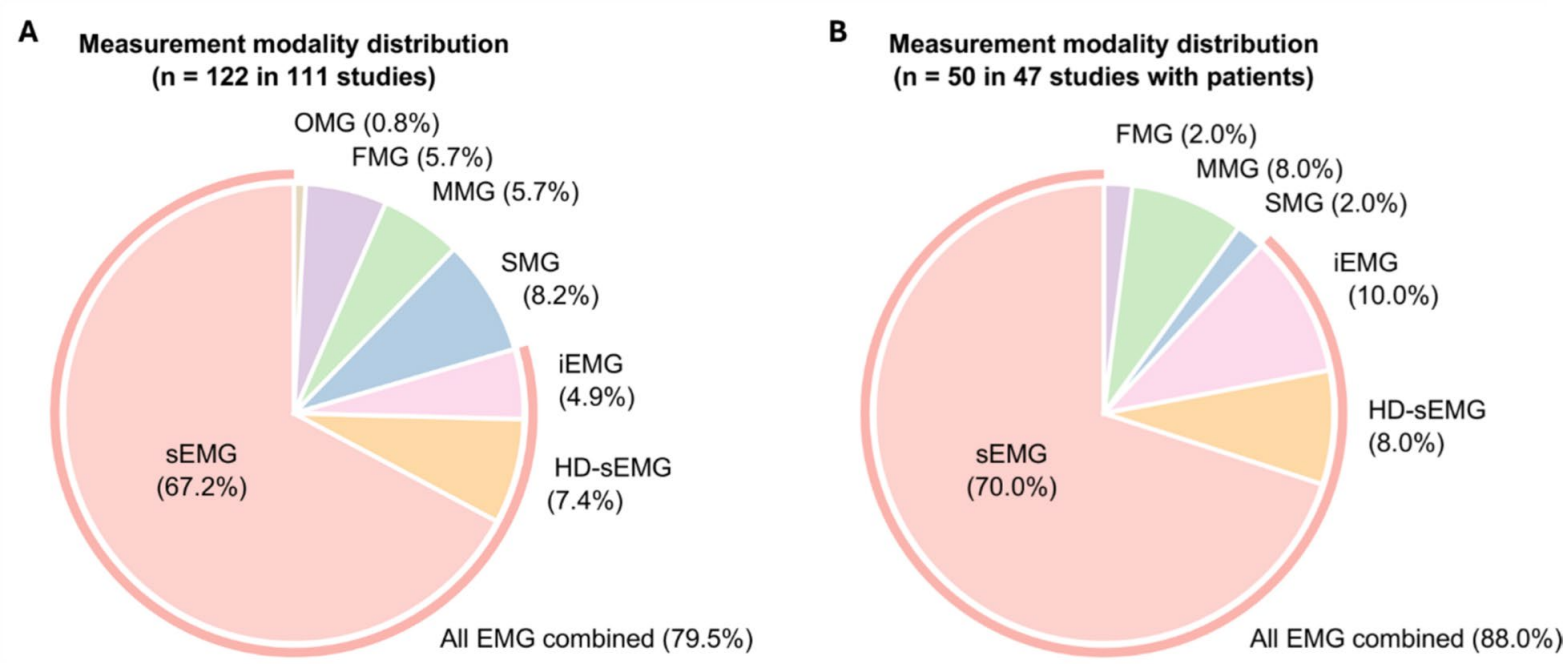
Distribution of measurement modalities used in the selected studies. **A** Among total 122 measurement modalities used across 111 studies, surface electromyography (sEMG) was the most prevalent modality, accounting for 67.2% of the total measurement modalities. Other modalities included sonomyography (SMG; 8.2%), high-density sEMG (HD-sEMG; 7.4%), mechanomyography (MMG; 5.7%), force myography (FMG; 5.7%), intramuscular EMG (iEMG; 4.9%), and optomyography (OMG; 0.8%). **B** Among total 50 measurement modalities used across 47 studies with patients, sEMG was the most dominant modality, accounting for 70.0%. Other modalities included iEMG (10.0%), HD-sEMG (8.0%), MMG (8.0%), SMG (2.0%), FMG (2.0%), and OMG (0.0%)

Various measurement modalities were used to measure myographic signals (Fig. 3), including surface electromyography (sEMG), intramuscular EMG (iEMG), high-density sEMG (HD-sEMG), sonomyography (SMG), mechanomyography (MMG), force myography (FMG), and optomyography (OMG). Among these modalities, EMG was the most frequently used measurement modality in all the reviewed studies (Fig. 3A), which accounts for 79.5% including sEMG (67.2%) [15–20, 22–25, 28, 30, 38–40, 48, 50–68, 70, 72–74, 76–80, 82–89, 92, 93, 95, 96, 98, 99, 101–103, 105–122, 124–135], HD-sEMG (7.4%) [15, 17, 50, 67, 84, 86, 101, 120, 133], and iEMG (4.9%) [20, 27, 29, 31, 90, 123]. This is followed by SMG (8.2%) [21, 46, 57, 68, 79–81, 89, 91, 100], MMG (5.7%) [26, 32, 33, 55, 94, 104, 134], FMG (5.7%) [49, 52, 69, 71, 97, 107, 127], and OMG (0.8%) [75]. Most measurement modalities were also used in the studies including patient group (Fig. 3B). There was a notable increase in the dominance of EMG by 7.5–2.8% from sEMG [15–19, 22–25, 28, 30, 108–122, 124–135], 0.6% from HD-sEMG [15, 17, 120, 133], and 5.1% from iEMG [20, 27, 29, 31, 123]—and of MMG by 2.3% [26, 32, 33, 134], while other measurement modalities including SMG (2.0%) [21], FMG (2.0%) [127], and OMG (0.0%) were less frequently used.

**Fig. 4:**
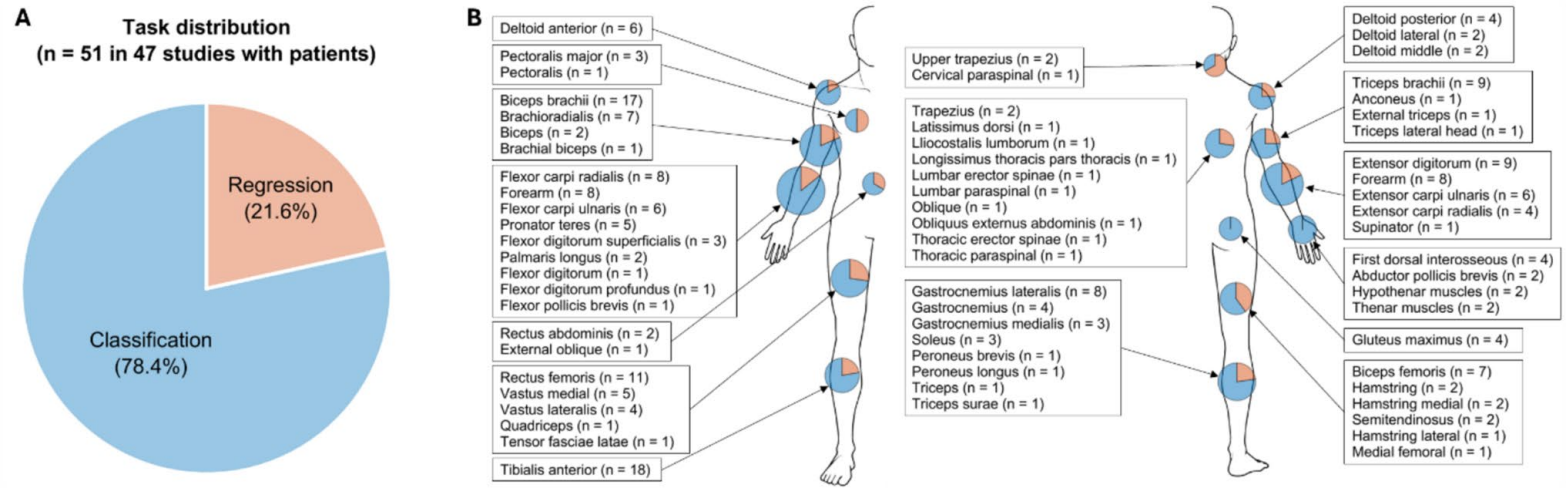
Applications and muscle group locations in the 47 studies involving patients. **A** Distribution or task. Among total 51 tasks reported, classification tasks accounted for 78.4% and regression tasks for 21.6%. Note that some studies addressed both classification and regression tasks. **B** Muscle group locations. Task proportions are depicted on a body schematic, with blue indicating classification tasks and red indicating regression tasks

The patient-involved studies employed myographic signals with AI models mainly to perform classification and regression (i.e., prediction) applications, while the portion of such tasks varied by locations (Fig. 4). Specifically, 78.4% of models in the studies focused on classification (Fig. 4A) to identify various tasks including gestures [15, 16, 23, 112, 120, 122, 127, 133], movements or activities [17–19, 21, 22, 24, 110, 111, 116–118, 126], and clinical conditions such as diagnosis [27–32, 109, 115, 119, 123, 124, 131, 132] and severity [33, 125]. The regression task was also conducted in 21.6% of the studies to predict assessment score [111, 121], joint angle or torque [25, 26, 135], and muscle activation level or EMG values [129, 130]. In addition, these tasks were applied to various muscles located in peripheral upper and lower limbs as well as neck and torso (Fig. 4B). Overall, lower limb was more frequently investigated compared to upper limb, and specifically at the ankle. Specifically, among the 47 studies that included patient populations, the tibialis anterior muscle was the most frequently assessed, appearing in 36.2% [19–21, 25, 29, 109, 114, 116–118, 123–125, 128–131], followed by the biceps brachii in 34.0% [17, 20, 21, 29–32, 108–111, 116, 121, 126, 128, 130], the rectus femoris in 21.3% [18, 29, 109, 114, 116, 117, 130, 131, 134, 135], the extensor digitorum in 17.0% [24, 111, 112, 119, 121, 122, 127, 132], the triceps brachii in 17.0% [17, 32, 108, 110, 111, 116, 121, 126], and the flexor carpi radialis in 17.0% [23, 24, 33, 110, 122, 126, 127, 132].

### Sample demographics

**Fig. 5:**
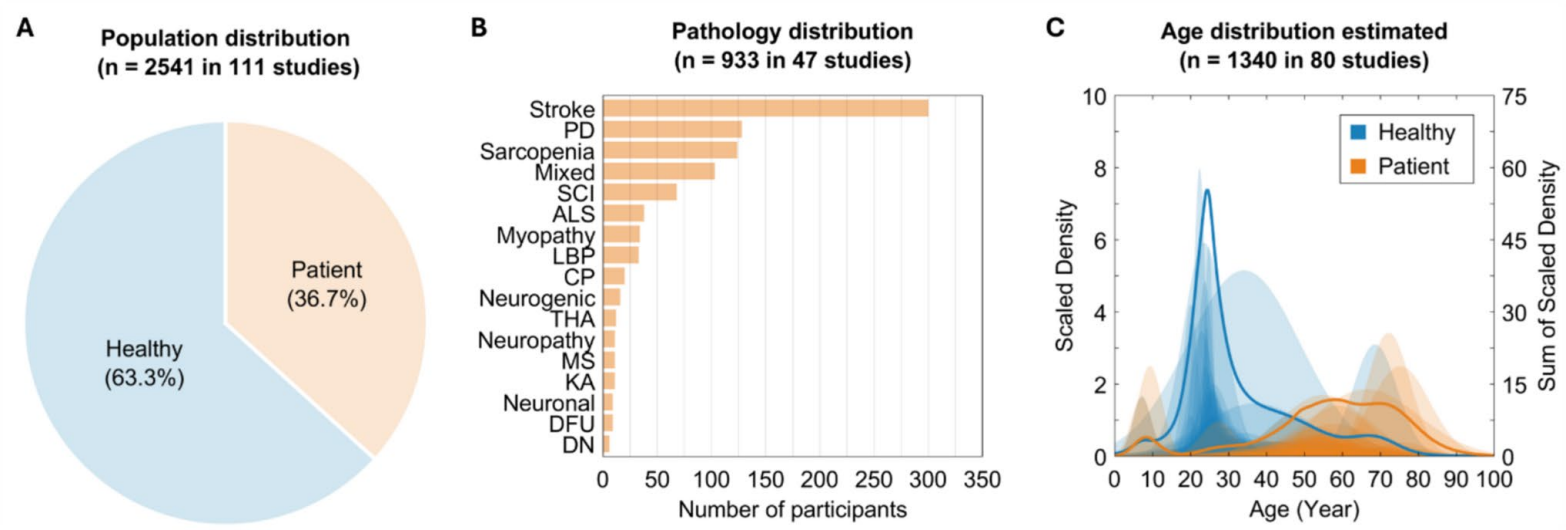
Demographic characteristics of study participants. (**A**) Distribution of healthy versus patient participants among total 2541 participants across 111 studies. (**B**) Distribution of pathology among total 933 participants in the 47 studies with patients. PD, Parkinson’s disease; Mixed, radiculopathy, polymyositis, muscular dystrophy, peripheral nerve injuries, normal pressure hydrocephalus, and stroke; SCI, spinal cord injury; ALS, amyotrophic lateral sclerosis; LBP, low back pain; CP, cerebral palsy; THA, total hip arthroplasty; MS, multiple sclerosis; KA, knee abnormalities; DFU, diabetic neuropathy with ulceration; and DN, diabetic neuropathic. (**C**) Scaled density plot of the estimated age distribution across 80 studies with a total of 1340 participants. The healthy group (blue) is predominantly younger, whereas the patient group (orange) exhibits a broader age range with a slight increase in density among older participants

The total sample size was 2,541 in the selected 111 studies, and the number of patient participants was 933 (36.7%) from the 47 patient-involved studies [15–33, 108–135] (Fig. 5A). Among the 47 studies that recruited patient participants, 14 studies (29.8%) aimed to balance the number of healthy and patient participants [21, 22, 25, 27, 28, 31, 108, 111, 114, 130–132, 134, 135]. Meanwhile, 16 studies (34.0%) focused exclusively on patient participants while applying various AI models with myographic signals [15, 17, 19, 20, 23, 24, 26, 33, 117, 118, 120, 121, 125–128].

When looking at the pathology distribution (Fig. 5B), stroke (32.2%; n = 300 participants in 16 studies [16, 18, 23, 24, 33, 108, 111, 113, 115, 116, 118, 121, 122, 126, 127, 129]) was the most dominant, followed by PD (13.7%; n = 128 participants in three studies [22, 32, 128]), sarcopenia (13.3%; n = 124 participants in three studies [125, 131, 132]), SCI (7.3%; n = 68 participants in ten studies [15, 17, 19, 25, 26, 110, 112, 120, 130, 133]), ALS (4.1%; n = 38 participants in four studies [21, 27, 119, 123]), myopathy (3.6%; n = 34 participants in six studies [27–31, 123]), low back pain (LBP; 3.5%; n = 33 participants in one study [109]), CP (2.1%; n = 20 participants in one study [117]), neurogenic (1.7%; n = 16 participants in two studies [29, 31]), total hip arthroplasty (THA; 1.3%; n = 12 participants in one study [114]), neuropathy (1.2%; n = 11 participants in one study [28]), MS (1.2%; n = 11 participants in one study [134]), knee abnormality (KA; 1.2%; n = 11 participants in one study [135]), neuronal (1.0%; n = 9 participants in one study [30]), diabetic foot ulcer (DFU; 1.0%; n = 9 participants in one study [124]), and diabetic nephropathy (DN; 0.6%; n = 6 participants in one study [124]).

Interestingly, the age distribution for healthy participants was highly focused on the young adults aged 20–30 years old (Fig. 5C), while the age of patient participants was more broadly distributed as supported, in part, by the various target clinical participants (e.g., CP, SCI, stroke, neurodegenerative diseases), nevertheless, more focused on the middle age group (e.g., over 40 years old). Overall, discrepancy between the age distribution, both within and across studies, between healthy versus patient participants was evident.

**Fig. 6:**
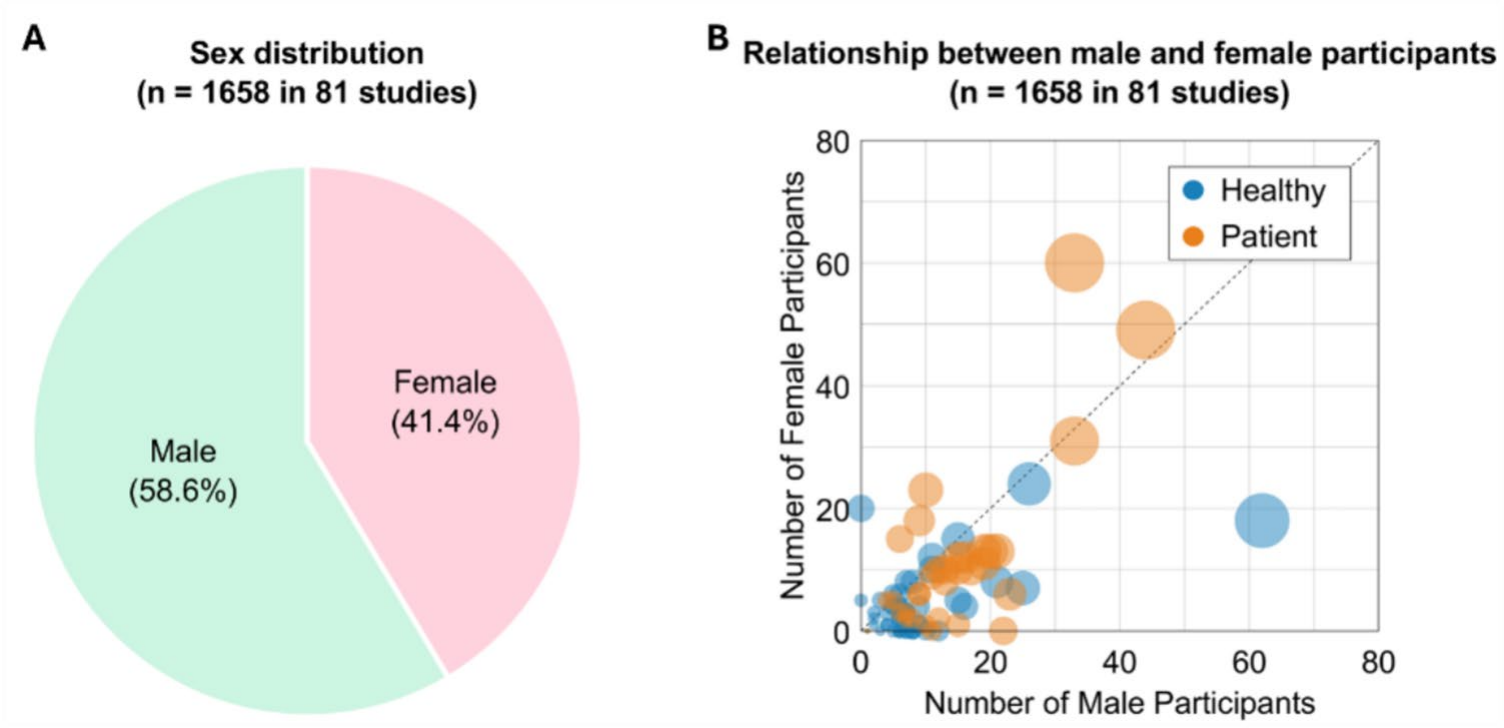
(**A**) Distribution of sex among total 1,658 participants in 81 studies where the number of both male and female participants was reported. (**B**) Relationship between male and female participants in the selected studies. Each circle represents an individual study, with circle size indicating total participant count. Blue and orange represent healthy and patient groups, respectively

The 81 studies that reported the number of male and female participants [15–17, 19, 21, 23, 27–33, 39, 40, 46, 48, 52–57, 59–65, 68, 69, 71–77, 79–91, 93, 94, 97, 100, 102–106, 108, 110, 111, 116–127, 130–132, 134, 135] recruited more male participants (n = 971; 58.6%) than female participants (n = 687; 41.4%) as a whole (Fig. 6A). Specifically, 26.1% recruited a relatively balanced number of male and female participants, range of the female-to-male ratio of 40–60% [16, 17, 21, 27, 29, 33, 40, 46, 59, 62, 64, 69, 74, 75, 84, 87, 89, 97, 102–104, 106, 116, 117, 122, 123, 125, 131, 134]. There were also several studies with more female participants, range of the female-to-male ratio of 50–100% [15, 17–24, 26, 27, 29–33, 108, 109, 112, 114, 117, 118, 120, 121, 123–128, 130, 132]. Relationship between the number of male and female participants, however, revealed that in general even at an individual study level, the samples were biased towards more male participants (Fig. 6B).

**Fig. 7:**
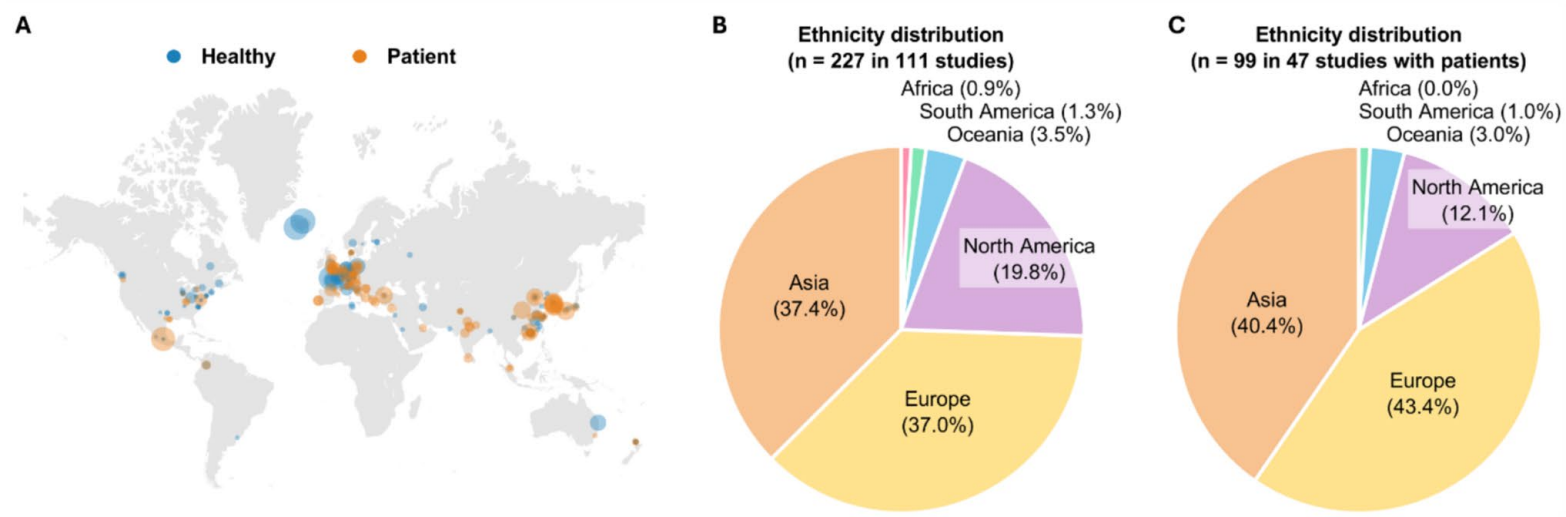
Distribution of geographic location and ethnicity based on the authors’ affiliations in the selected studies. (**A**) Global distribution, highlighting a higher concentration of the studies in North America, Europe, and Asia, with each circle representing an individual study and sized according to total participant count. Blue and orange represent healthy and patient groups, respectively. Distribution of ethnicity from 227 locations in 111 studies (**B**) and from 99 locations in 47 studies with patients (**C**). Note that multiple author affiliations per study result in a higher number of locations than studies

Affiliations of the authors in the selected studies—an estimate of ethnicity (or nationality) in the participant population—were highly biased to only few regions across the globe: mainly from northeastern Asia, western Europe, and north America (Fig. 7A). Among the 227 different countries in the 111 studies (Fig. 7B), 37.4% were from Asia [15, 18, 20, 21, 23–26, 28, 31, 38–40, 48, 50, 51, 53, 54, 56, 57, 59, 60, 63, 67, 70, 75, 77, 78, 82, 88, 90, 92–95, 101, 105–108, 111, 112, 115, 116, 118, 121–124, 126, 127, 129, 131, 132, 135], and 37.0% were from Europe [16, 17, 19, 23–25, 27, 29, 30, 32, 33, 50, 52, 55, 57–61, 63, 64, 68, 72, 73, 81, 83, 86, 87, 90, 96–99, 102, 103, 109, 114, 116–120, 125, 129, 133, 134]. North America accounted for 19.8% [15, 19, 22, 24, 46, 49, 66, 69–71, 73, 74, 76, 79, 80, 84–86, 89, 91, 93, 100, 104, 110, 112, 120, 128, 130], followed by Oceania for 3.5% [23, 26, 59, 62, 72, 86, 90, 118], South America for 1.3% [65, 82, 113], and Africa for 0.9% [97]. When considering the 47 patient-involved studies only (Fig. 7C), Europe had the largest proportion at 43.4% [16, 17, 19, 23–25, 27, 29, 30, 32, 33, 109, 114, 116–120, 125, 129, 133, 134], followed by Asia at 40.4% [15, 18, 20, 21, 23–26, 28, 31, 108, 111, 112, 115, 116, 118, 121–124, 126, 127, 129, 131, 132, 135], North America at 12.1% [15, 19, 22, 24, 110, 112, 120, 128, 130], Oceania at 3.0% [23, 26, 118], South America at 1.0% [113], and Africa at 0.0%.

### AI model

**Fig. 8:**
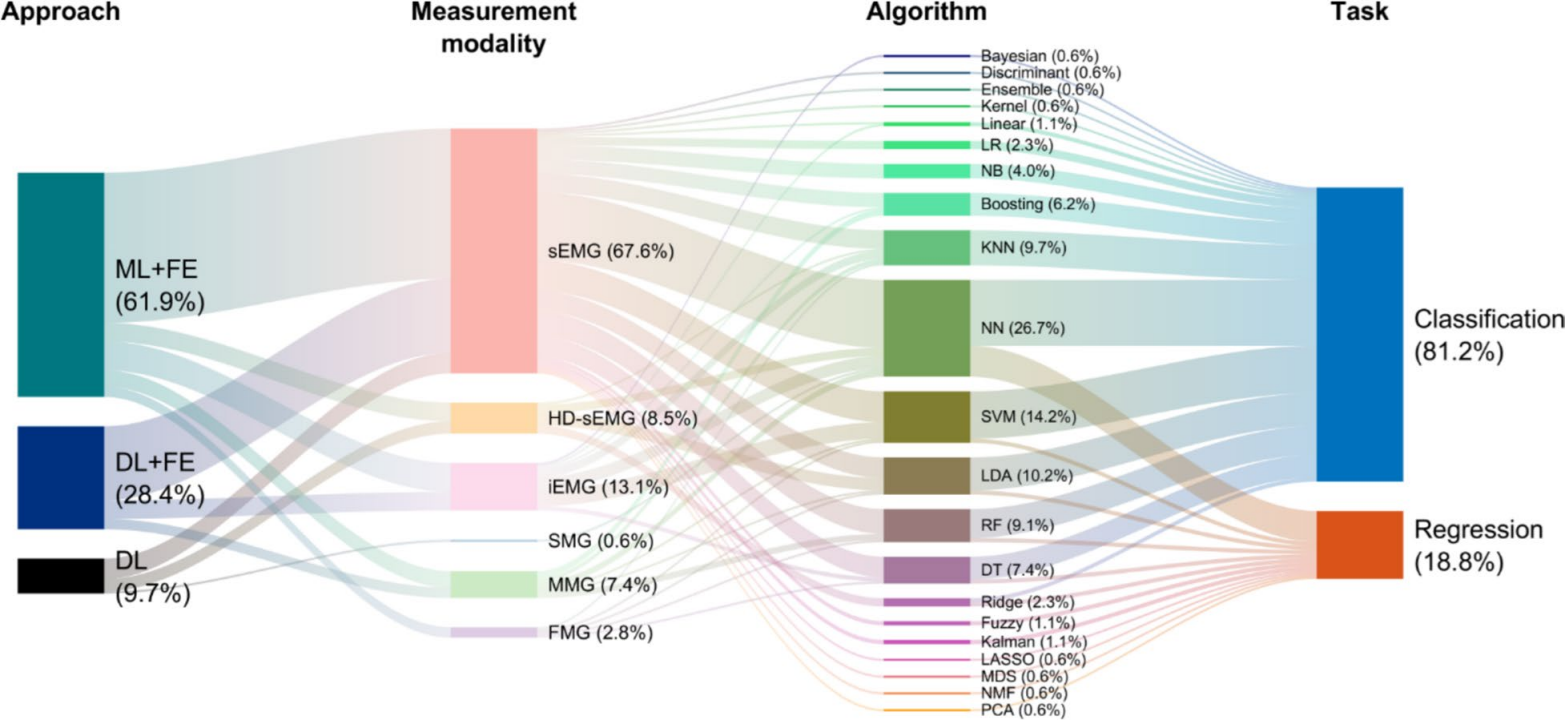
Sankey diagram illustrating the relationships between approach, measurement modality, algorithm, and application reported in the 47 studies with patients. This diagram visualizes the interconnections between different model approaches, including machine learning with feature engineering (ML + FE), deep learning with feature engineering (DL + FE), and deep learning without feature engineering (DL), and their applications in classification and regression tasks. sEMG, surface electromyography; HD-sEMG, high-density sEMG; iEMG, intramuscular EMG; SMG, sonomyography; MMG, mechanomyography; FMG, force myography; LR, linear regression; NB, naïve bayes; KNN, k-nearest neighbors; NN, neural network; SVM, support vector machine; LDA, linear discriminant analysis; RF, random forest; DT, decision tree; LASSO, least absolute shrinkage and selection operator; MDS, multidimensional scaling; NMF, non-negative matrix factorization; PCA, principal component analysis

Various learning approaches and algorithms were used (Fig. 8). Among the patient-involved studies, 90.3% employed various feature extraction and feature selection methods to utilize the measured myographic signals in different AI models [15, 17–20, 23–33, 108–111, 113, 115, 116, 118–127, 129–134]. Among the 28 studies utilizing neural networks (NNs), 64.3% further incorporated separate feature engineering techniques—while not necessary for deep learning models—for their analysis [24–28, 31, 32, 111, 113, 115, 118, 121, 123, 125, 126, 129–131]. In contrast, 35.7% used preprocessed myographic signals as direct input without additional feature engineering [16, 21–23, 112, 114, 117, 128, 133, 135].

In the 33 studies employing traditional machine learning methods for classification or regression tasks, 66.7% of the studies utilized sEMG [18, 19, 23, 25, 30, 108–111, 113, 115, 116, 118–120, 122, 124, 125, 127, 130–132, 134], 12.1% applied HD-sEMG [15, 17, 120, 133], and 15.2% incorporated iEMG [20, 27, 29, 31, 123] as their primary measurement modality. Additionally, 9.1% investigated MMG [32, 33, 134], 3.0% focused on FMG [127], and 0% explored SMG. These studies mainly relied on conventional machine learning algorithms, such as k-nearest neighbors (KNN), linear discriminant analysis (LDA), support vector machine (SVM), decision tree (DT), or random forest (RF). In contrast, 28 studies that implemented deep learning techniques using NNs showed 75.0% focused on sEMG [16, 22–25, 28, 111–115, 117, 118, 121, 125, 126, 128–131, 135], 3.6% applied HD-sEMG [133], and 10.7% utilized iEMG [27, 31, 123]. Furthermore, 7.1% worked with MMG [26, 32], 3.6% with SMG [21], and 0% with FMG.

Among the 39 papers related to classification, a survey of various AI models revealed that 69.2% employed NNs [15, 16, 19, 21–24, 27, 28, 30–32, 111, 112, 114, 115, 117–119, 123–128, 131, 133], 38.5% used SVM [18–20, 27, 29, 31, 111, 115, 116, 123–125, 127, 132, 134], and 25.6% utilized LDA [17, 23, 110, 118–120, 122, 127, 133, 134]. In contrast, among the 11 papers related to regression, 90.9% applied NNs [25, 26, 111, 113, 117, 121, 129, 130, 133, 135], 9.1% employed Support Vector Regression (SVR) [111], and 9.1% used Linear Discriminant Analysis (LDA) [133]. It is important to note that the total number and percentages are higher due to the use of multiple sensors or models within a single paper.

## Discussion

In this review, we sought to provide evidence from current literature and state-of-the-art applications for whether using myographic signals with AI can offer better insights and performance in understanding and assessing motor impairments. Specifically, our goal was to conduct a scoping review of relevant works up to date using myographic signals with AI in terms of:

- *Target application* to determine whether the use of myographic signals indeed can provide more tailored insights into assessing motor impairment and/or allow for better performance of AI models compared to other measurement modalities such as IMU.
- *Sample demographics* to determine whether there exist any potential biases that may hinder the generalization of the developed/applied AI models, accounting for the heterogeneity in a particular clinical target population, or even broader populations in general.
- *AI model* to determine whether the type of the models being used have potential implications for generalizability to diverse contexts of application.

In the following sections, we review the main findings, discuss the limitations and remaining challenges, and propose potential solutions and future directions on the above aspects.

### Target application

In summary, we found that dominantly EMG, especially sEMG, is being used to acquire myographic signals (Fig. 3), where the measurement took place in various body parts or muscles (Fig. 4B), largely for classification tasks (Fig. 4A).

In agreement with our postulation, we found promising evidence demonstrating that myographic signals can provide more direct insight into the muscle activity over other measurement modalities such as IMU that primarily capture movement-initiated patterns. Wang et al. (2020) employed both kinematic and EMG data to classify normal and pathological movement patterns [111]. While the classification accuracy using kinematic data reached 92.82% with an SVM model—slightly outperforming EMG-based accuracy (89.35%)—the EMG data achieved higher accuracy with artificial neural network method (BPNN) (90.74%) and RF (89.12%) models compared to kinematic-based accuracy (88.19% and 88.66%, respectively) (Table 3). In Song et al. (2022), focused on classifying 12 hand movements, the classification accuracy was only 47.8% when using IMU data alone [127]. In contrast, using either FMG or EMG alone resulted in significantly higher accuracies of 63.2% and 69.6%, respectively. Furthermore, most of the reviewed studies demonstrated that the utilization of myographic signals with AI offers possibilities in: (1) the diagnosis of neuromuscular diseases, including myopathy [27–31, 123], neurogenic [29, 31], ALS [20, 21, 27, 119, 123], and DN [124]; (2) the detection of abnormal muscle activity patterns from resting EMG signals [20] and from B-mode ultrasound images [91]; and (3) the assessment of the physical activity level in MS patients [134] and of the severity level of sarcopenia in older adults [125, 131, 132]. Additionally, we found some studies in the context of human–machine interfaces (HMIs), to detect/recognize user intentions [77, 136].

**Table 3:** The performance differences between myographic signals and data of other modalities

Study	Tasks	Evaluation	Performance	
Li et al. (2017)	Regression	Correlation (Fugl-Meyer assessment and evaluation indicator)	EMG	0.3563 (DTW), 0.6672 (PCC)
			IMU	0.7977 (DTW), 0.8736 (PCC)
			Fusion	0.7781 (DTW), 0.8780 (PCC)
Huo et al. (2020)	Classification	Accuracy (Healthy vs UPDRS scores (1–3))	MMG	76.3% (Bradykinesia), 81.5% (Tremors)
			IMU	82.7%
			Force sensor	81.7% (Elbow), 82.5% (Wrist)
			Fusion	85.4%
Wang et al. (2020)	Classification	Accuracy (Healthy vs Pathological)	EMG	NN: 90.74%, RF: 89.12%, SVM: 89.35%
			Kinematic	NN: 88.19%, RF: 88.66%, SVM: 92.82%
			Fusion	NN: 94.21%, RF: 93.89%, SVM: 96.06%
	Regression	Correlation (Fugl-Meyer assessment and predicted score)	EMG	− 0.69
			Kinematics	− 0.72
			Fusion	− 0.87
Meng et al. (2021)	Classification	Accuracy (Daily activities)	EMG	90.87 ± 3.41% (Healthy), 73.77 ± 3.73% (Stroke), 84.09 ± 3.33% (All), 76.34 ± 3.75% (Train: healthy, test: stroke)
			Gyroscope	94.54 ± 2.06% (Healthy), 75.88 ± 5.53% (Stroke), 87.95 ± 2.79% (All), 76.82 ± 5.55% (Train: healthy, test: stroke)
			Accelerometer	96.43 ± 2.35% (Healthy), 94.05 ± 3.10% (Stroke), 95.84 ± 1.75% (All), 77.89 ± 4.81% (Train: healthy, test: stroke)
			Fusion	96.53 ± 2.36% (Healthy), 94.22 ± 2.63% (Stroke), 96.56 ± 1.55% (All), 82.47 ± 4.18% (Train: healthy, test: stroke)
Zhou et al. (2021)	Classification	Accuracy (Eight hand movement)	EMG	Normal subjects: 88.71 ± 2.79% Post stroke patients: 83 ± 8.21%
			Kinematic	Normal subjects: 84.49 ± 6.77% Post stroke patients: 84.71 ± 4.54%
			Feature fusion	Normal subjects: 96.90 ± 1.81% Post stroke patients: 96.43 ± 3.83%
			Decision fusion	Normal subjects: 93.91 ± 2.57% Post stroke patients: 91.18 ± 5.50%
Song et al. (2022)	Classification	Accuracy (12 hand movements)	FMG	63.20%
			EMG	69.60%
			IMU	47.80%
			Fusion	81.00%
Hou et al. (2023)	Classification	AUC (Detection of freezing of gait)	EMG	0.78
			ACC	0.71
			EEG	0.75
			Fusion	0.85

*AUC, area under the curve; DFU, diabetic neuropathy with ulceration; DN, diabetic neuropathic; DTW, dynamic time warping correlation; PCC, Pearson’s correlation coefficient

Interestingly, we also found studies that demonstrate the use of myographic signals in combination with other motion sensor modalities (e.g., kinematics, accelerometer, gyroscope, IMU) offering better performance, compared to just using myographic signals or motion signals, in classification [32, 111, 116, 128] and emulating clinical scores [111]. Zhou et al. (2021) demonstrated that for classifying eight hand gestures in healthy individuals, combining EMG and kinematic features resulted in an accuracy of 96.90 ± 1.81%, which was significantly higher than using EMG features alone (88.71 ± 2.79%) [122]. Similarly, in stroke patients, the combined modality achieved 96.43 ± 3.83%, compared to 83.00 ± 8.21% using only EMG. Wang et al. (2020) found that the correlation between predicted and actual upper limb function improved from − 0.69 with EMG alone to − 0.87 when kinematic features were included [111]. These findings suggest that different sensor modalities such as myographic and motion signals can complement each other for better (more accurate, robust) performance [32, 122].

Despite the promising evidence for the unique benefits that myographic signals can offer when used with AI for motor impairment assessment, there are remaining challenges. Owing to the inherent characteristics of EMG signals—capturing electrical action potential conducted through nerves—robust acquisition is relatively difficult, compared to other measurement modalities. For instance, signal quality can be significantly influenced by non-physiological factors such as sensor size, placement location, the presence of body hair or dead skin cells, as well as sweating and movement-induced changes in sensor adherence during the experiment [104, 137]. In particular, non-stationarity of the signal [138, 139], as well as natural redundancy in how the same motor task can be performed using different motor commands [140, 141], induce variations (e.g., spatiotemporal, time and/or frequency domain) to the input for an AI model [142] and thus likely degrade the model performance (training vs unseen dataset), especially for applications that are intended to be used for long period of time (e.g., across days or longitudinal) [143, 144]. In addition, due to the unique information based on frequency contents [145], acquisition requires relatively high sampling rate of ≥ 1 kHz (cf. usually ~ 100 Hz for IMU), which demands for more power consumption and computational resources (e.g., processing, data storage). It is also worth noting that while other myographic signals based on SMG, MMG, FMG, and OMG—capturing physical changes in response to muscle excitation—can potentially overcome some of the limitations and may provide more robust inputs for AI models, these modalities also come with their own challenges, for example, related to signal-to-noise ratio and susceptibility to motion artifacts [146, 147].

Advances in sensor (e.g., materials, form factors, power/resource management) [148], signal processing techniques [145], and robust AI algorithm development [14] will be essential for overcoming these challenges. Another noteworthy future direction that this scoping shed light on is sensor fusion approach. Although the current review focused on the use of myographic signals in comparison to and implication with respect to motion data (e.g., IMU), other measurement modalities such as ECG, PPG, EDA, and/or EEG [149, 150] may provide additional, more comprehensive yet tailored insights into the physiological state underlying a specific motor impairment at an individual-specific, systemic level, complementing the unique motor perspective conferred by myographic signals, as supported in part by Hou et al. (2023) showing that for detecting freezing of gait, the area under the curve increased from 0.78 with EMG alone to 0.85 with EMG combined with accelerometry and EEG signals [128]. Please refer to Sect. 4.4. for discussions on the implications in terms of implementation.

### Sample demographics

In summary, significant demographic biases, particularly regarding age, sex, ethnicity, and pathology, were observed in the reviewed studies (Figs. 5, 6 and 7).

Given the established anatomical, biomechanical, and physiological differences across diverse populations, these biases would likely introduce variations in not only the input myographic signals that any AI model is being trained with but also the (often latent) features being captured/learned. Consequently, such biases may limit the generalizability of the developed application to broader target and ultimately hinder the clinical translation. Implication of such differences across diverse populations is increasingly gaining attention in science, probably due to heterogeneity across individuals. For example, there are measurable sex-/ethnicity-based differences in inherent neuromuscular performance such as body composition (e.g., muscle mass and fat distribution) [151–153], muscle strength and power [152, 154, 155], muscle architecture (e.g., fascicle length, pennation angle, muscle thickness) [156, 157], and muscle fiber characteristics (e.g., fiber type, cross-section area) [158, 159]. Furthermore, age-related changes, exercise adaptations, and pathological conditions can lead to even greater diversity in neuromuscular mechanisms including motor unit firing behaviors (e.g., firing rate, recruitment) [160–162], muscle fiber conduction velocity [163–165], muscular changes in size (e.g., atrophy, hypertrophy) [158, 166, 167], architecture [168–170], material properties (e.g., composition of adipose tissue and fibrous collagen in extracellular matrix) [171–173], and fiber type composition [158, 164, 174], and muscle coordination [175–177]. In addition, lifestyle-related factors such as physical activity, nutrition, and comorbidities may further introduce the variability of myographic signals [178–180].

The inclusion of diverse populations is essential to enhance the generalizability of research findings across a wide range of individuals and contexts. However, it is well-acknowledged that acquisition of such a comprehensive dataset practically is nearly impossible for any individual investigator or research lab, especially for clinical population [181]. Such challenge can be overcome with effort as a community, such as openly sharing data (e.g., repository, database), which, encouragingly, seems to be the recent trend in many disciplines [182–184]. In order to maximize the potential of such combined effort, standardized protocols for data acquisition and processing are essential [182]. Moreover, the integration of advanced techniques, such as data augmentation leveraging generative AI models [185, 186], may provide valuable insights. Nevertheless, it is essential to carefully consider the methodological implications and caveats associated with these approaches, including potential biases and limitations in data quality, validity, and reliability. Additionally, while longitudinal, real-world tracking of quantitative motor impairment-related data, including myographic signals, is becoming more accessible with the advances in wearable sensor and remote monitoring techniques [187, 188], a care must be taken in protecting healthy-related and privacy information [184].

### AI model

In summary, we found that machine learning with feature engineering is the most dominant category of AI models that are being used with myographic signals for clinical applications (Fig. 8). It was interesting to note that deep learning models, which by virtue does not necessarily require a priori definition of specific features to learn from the input dataset [189], were more often used with feature engineering. We also found that neural network is the most widely used model type/architecture, where, in many cases, various models were used in one study to compare the performances.

The performance of an AI model trained with relatively small data (e.g., sample size) with respective to model complexity (e.g., number of features or parameters) as well as for particular purpose (e.g., classification or prediction) will likely not generalized to other data set or application [190]. While feature engineering can improve the performance of AI models, it may potentially increase the risk of overfitting [191]. In addition to ensuring the diversity in the input data/sample discussed above (in Sect. 4.2), there are approaches that can be adopted to improve the generalizability and robustness of the AI model for broader contexts of application. For example, transfer learning is a scheme that leverages cross-domain techniques to generalize a model pre-trained with initial source data/domain to newly recorded target data/domain without the necessity for complete retraining or recalibration of the model [192]. Successful examples, in the context of hand gesture classification, include retaining accuracy across EMG data measured from different users, sensor locations, and days (within the same user) [193]. Alternatively, various model-specific/agnostic explainable AI techniques and tools applied at local/global scope (e.g., SHapley Additive exPlanations (SHAP) or Local Interpretable Model-Agnostic Explanations (LIME) [194]), may allow for identification of key features that can be adapted to guide and facilitate the generalization of a particular AI model to a different set of data or application (e.g., patient, clinical target). Compared to other disciplines and applications, such approaches have been rarely applied for AI models using myographic signals, especially for clinical target [195, 196].

While our initial intent was to also investigate, among the studies reviewed, the effect of model complexity, such as by examining its correlation with the sample size and/or performance, we could not find a single, suitable measure for model complexity that can be commonly applied to all models reviewed [197]. Moreover, many studies did not report the basic information about the AI model (e.g., architecture, size) from which we can infer the complexity [24, 27, 126]. At the minimum, if not tested explicitly, it is encouraged that such information is provided to aid in gauging the generalizability of the model. Furthermore, we assert that the development of universal/versatile measures and means to evaluate the model complexity is needed, which, analogous to the established power analysis tools for statistics, can inform and ultimately guide the selection of type, size, structure/architecture of AI models to use.

### Clinical translation

Ultimately, we emphasize the following two important aspects to be considered, and implemented, for any application using myographic signals with AI to find its place in the real world, that is, deployed in the field (e.g., clinics, bedsides, home) and adopted by the users (e.g., clinicians, patients, and their caregivers). Firstly, the technology as the entire package should be user-friendly. For example, the sensor/device should be easy to “do-on-and-off” (i.e., easily/quickly placed without much care), requiring minimal (ideally single) placement and setup. In case of multi-modal measurements or sensor fusion, recent advancements in sensor integration technology appear to be promising to pack multiple sensors onto a single, smaller chip [149]. The control/software interface should also be simple and intuitive, requiring minimal to no technical knowledge and/or training for clinicians and patients to easily use [198]. Secondly, the model outcomes should provide clinically relevant information. Whether providing a very close link (e.g., strong correlation) to the conventional clinical assessment measures or newly devised outcome metrics, the information gathered/synthesized must readily translate to what clinicians currently use and correspond to what the patient experiences in everyday life [199, 200]. Li et al. (2017) measured muscle activity using EMG and IMU modalities while participants performed 11 different tasks [108]. The collected data were then used to compute evaluation indicators through supervised or unsupervised algorithms, which were subsequently compared with clinician-provided Fugl-Meyer assessment scores. The results demonstrated a strong correlation, with a Pearson’s correlation coefficient of 0.88 and a dynamic time warping correlation of 0.78. These findings indicate that values predicted through myographic signals and AI models can closely reflect traditional clinical assessments. Although the clinical application of myographic signals still requires proper electrode placement, clinician training in the use of such modalities, and standardized analysis methodologies, the integration of AI models has the potential to reduce evaluation time, minimize inter-clinician variability, improve sensitivity to subtle changes, and enhance comparability in longitudinal studies [137].

## Conclusion

In conclusion, this scoping review highlights the promising application of myographic signals with AI in understanding and assessing motor impairments. Through an extensive search of the Scopus and PubMed databases, our analysis demonstrated that sEMG is the predominant measurement modality for acquiring myographic signals, mainly used for classification tasks, and that machine learning with feature engineering is the most common AI approach employed in clinical applications, including identification of neuromuscular diseases. To promote the practical adoption of various myographic signals in clinical settings, strategies to improve data quality during the measurement process are essential. Given that the use of current myographic measurement modalities requires extensive training for clinicians [121], future research should focus on reducing the complexity and minimizing inconvenience of use. This may include the development of more user-friendly sensor modalities, strategies to reduce placement variability by narrowing the acceptable sensor attachment area, or the establishment of standardized analytical methodologies to minimize the need for training for clinicians and patients. Additionally, to enable broader applicability of the developed models across various domains, it is essential to provide detailed descriptions, including the model architecture, number of layers, activation functions, optimization algorithms, model parameters (total and trainable), weight initialization methods, and input and output sizes [197]. Moreover, our findings showed significant demographic biases within and across studies, suggesting the need for more diverse and representative datasets. To this end, future studies should recruit participants from a wider range of disease groups and diverse ethnic backgrounds, while ensuring balanced gender distribution, equal sample sizes between control and experimental groups, and consistent age ranges across groups. We also discussed two important aspects to translate this effort of using myographic signals with AI into real-world clinical practice. Ultimately, we believe that myographic signals, given the essential physiological information it conveys at high spatial and temporal resolution, combined with AI approaches that robust and accurate performance offers great potential for precision medicine in the context of motor impairment assessment.
